# Kitson versus Playfair

**Published:** 1896-04-04

**Authors:** 


### The Hospital

An Institutional Journal of

The Medical Sciences and Hospital Administration, with

AN EXTRA NURSING SUPPLEMENT.

Vol. XX.—No. 497.] [April 4, 1896.

### Kitson versus Playfair.

Many points arose in the course of this case which are full of interest, and it may be added, full of lessons, to the medical profession. Among them, however, two stand out pre-eminent—the question of privilege, on which the verdict turned, and the question of breach of professional confidence, which, although it had nothing to do with the legal aspect of the case, undoubtedly influenced the jury in the damages they awarded.

Many erroneous notions are current as to the obligation of secrecy which is imagined to lie upon all medical men in regard to knowledge obtained in the course of their professional duties. It is thought by many that the confessions of the consulting-room are as sacred as, in some countries, are the revelations of the confessional. But this is not so. In some countries a doctor who for any cause breaks professional confidences is amenable to the law, and even in a court of justice he can plead privilege and refuse to declare what has been told him by his patient. But in England things are different, and the veil of secrecy which covers what is told to a doctor is entirely a matter of custom and of tradition. Few things show more markedly the confidence which the general public have in the honour of the medical profession than the way in which, depending merely on professional custom and the honour of the individual, they entrust to medical men details of their lives which, if whispered abroad, would bring upon them shame and misery, and in many cases might place them within the grip of the law.

It becomes then a matter of the extremest interest to determine under what circumstances, if any, this seal of secrecy may properly be broken. In regard to this there was some difference of opinion between the various medical witnesses in the case. On the one hand, Dr. Herbert Spencer, while not claiming that there was any rule which appertained to the medical profession other than the general rule which bound all honourable professional men in respect to the confidences of their clients, only allowed two exceptions to the general obligation that information obtained during attendance should be considered confidential. One would, he said, have to divulge a professional secret if not divulging it would make one accessory to a crime, and he thought that in a court of law a man was bound to divulge such secrets.

According to Sir John Williams, the exceptions to the rule came in where there was a higher claim, as for example, where a man had to give evidence in a court of justice. A medical man was also obliged to divulge or inform the Public Prosecutor of any crime which had been committed, or which was intended to be committed, and the higher claim which a man's wife and children had upon him justified him in taking every measure to protect them.

Sir W. Broadbent also thought it was conceivable that it might be one's duty to reveal a professional secret under various circumstances.

The suggestion that things may be done for wife and family which would not be permissible in regard to others raises very large issues, as, in fact, was shown in this very case, and probably the best comment upon it is that made by the Judge, that it does not follow that there is no other way of protecting them.

As regards the law courts, there seems no doubt that a medical man's professional secrets are without protection, and that he must divulge what he knows in answer to questions at the direction of the judge. But that is a very different affair to disclosing professional secrets to any lawyer who may be getting up a case, and this is a distinction which should be borne in mind by every medical man; it is in the court alone that he can be made to disclose.

There remains, then, the question of breaking professional confidence by giving information to the Public Prosecutor in regard to attempted crime. As to this the medical profession have to thank Mr. Justice Hawkins for a very definite pronouncement. It has lately been far too much the fashion to maintain that it is the doctor's duty to act as jackal to the authorities, and to hold that when suspicious circumstances come to his knowledge they should at once be communicated to the coroner or the police. The summing-up in this case should, however, clear the air to a large extent in regard to this misconception.

The Judge's words are reported as follows: "He did not think that a general rule could be laid down, nor did he agree with some of the witnesses that a medical man should, as a rule, violate professional confidence in certain cases. For instance, a medical man might have been attending a young woman whom he thought was trying to bring about abortion, but was he bound in consequence of this to go to the Public Prosecutor and say, 'I have been attending this young woman; she has been trying to effect abortion; she is getting better, and in a few days will be out again, so I think I ought to put you on your guard'? He did not think that there could be a general rule in the medical profession that where a medical man thought that a crime had been committed, or was about to be committed, he should communicate with the Public Prosecutor, though no doubt there were cases in which he might say that it was his obvious duty to disclose what had come to his knowledge, such, for instance, as a case of murder or attempted murder; but it would be a monstrous thing to say that in all cases where a doctor had reason to suspect crime he should communicate with the Public Prosecutor."

All this brings us back to the plain and simple rule, that professional confidence should not be broken unless, under the direction of a judge, in a court of justice a witness is made to answer the questions put to him, or unless, as Dr. Spencer put it, the not divulging it would make one accessory to crime. We take it then that this case should be taken to heart by the profession as emphasising, even by a dictum from the judicial bench, that old tradition which dates back to the very origin of the art of medicine, that professional secrets should be held inviolate.

